# Cerebro-Spinal Meningitis

**Published:** 1872-08

**Authors:** S. B. Junkin

**Affiliations:** Millersburg, Ind.


### Correspondence.

### Cerebro-Spinal Meningitis.

MILLERSBURG, IND.

PROF. MINER,

DEAR SIR:—I take the liberty of sending you a report of treatment of cerebro-spinal meningitis which I have used with satisfactory results in my practice in Ohio in 1868–9, and during the past month in this locality, where it is now prevailing to a limited extent. The symptoms already published in the *Buffalo Medical Journal* are very full and complete, and I send you a copy of daily record of the last case treated, which resulted in complete recovery and embraces, in the main, the treatment that has been of most service to me in this disease.

April 11, 1872. Called to see J. S.'s daughter, aged eight years. Taken sick at school the 10th; complained of chilliness and pain in the head; came home and went to bed. The family physician called and prescribed for remitting fever. Patient continued to grow worse, became delirious, complained of pain in the head, occasional vomiting, pain in the bowels, until we were called at eleven A. M., as above stated.

Found the patient in convulsions. Administered chloroform sufficient to produce partial anæsthesia, put her in warm wet pack from the arms to the feet, applied ice to the head, gave full dose of sulph., morphia by applying it dry to the tongue. Convulsions controlled in about an hour, but there was a continued rolling of the head, oscillation and turning up of the eye-balls, the pupils dilating at times and again becoming normal and contracted; a tendency to throw the head back and burrow it in the pillow; and when allowed to come from under the influence of the anodyne an incessant tossing and throwing of the body from one side of the bed to the other.

℞ F. Ext. Hyoscyamus, 5 drops.
" Indian Hemp, 3 drops.
S. Every three hours in one-half teaspoonful of glycerine.

℞ Bromide of Potassa, gr. iij.
" Ammonia, " ss.
S. Give in solution every four hours.

℞ Morphine when the Hyoscyamus failed to quiet sufficiently.

Friday, 12th, nine A. M. Patient more quiet, but still unconscious; continued dilitation of the pupils, tossing of the head, &c., slight cough, accumulation in the bronchia. Sent for counsel.

Ten A. M. Patient more quiet; rational for a few minutes at a time, sufficient to recognize the parents when aroused.

Twelve M. Face flushed, threatened with spasms. Repeated the warm pack, increased the ice to the head and neck, applied sinapisms the whole length of the spine. In an hour she was again relieved so as to recognize friends when aroused, but sinking again into delirium and coma. Nervousness very well controlled.

Counsel arrived; agreed upon diagnosis and treatment.

Hydrarg. sub. muriate, grs. xii. Divide into three parts; give a powder every three hours.

Discontinued the iodide while giving the sub. muriate; continued the Hyoscyamus and Indian Hemp, ice to the head and morphine when necessary. Staid with the patient until twelve at night. Rested quiet most of the time; extremities quite cold; applied jugs of hot water.

Saturday, 13th, nine A. M. Bowels not moved; gave injections of beef tea and brandy; bowels moved well at eleven A. M.; continued to move frequently during the afternoon; fever and flushing again in the afternoon; continued the treatment; added bromide of potassa and ammonia.

Sunday, 14th, three o'clock in the morning. Febrile symptoms entirely controlled; pulse very weak; discontinued the potassa.

℞ Brandy, one-half teaspoonful every hour.
Sulph. of quinine, grs. iss.
Dover powder, grs. ss.

S. One powder every three hours.

Beef essences, *ad libitum*.

Three P. M. Bearing stimulants and tonics well; the heat in the head somewhat increased, but resting well and says she feels better; bowels moving less frequent.

Monday, 15th. Quiet night's rest; diarrhœa controlled; continued tonics and stimulants.

12 M. Condition unchanged, except indications of moisture; skin quite soft.

Tuesday, 16th. Rested well during early part of the night; fever came on in the after part; gave dover powder.

At nine A. M. fever subsided.

℞ Quinine, grs. iss.

Every three hours until three have been given.

Cough very troublesome; expectorating freely; no complaint of pain.

Wednesday, 17th, nine A. M. Patient rested well through the night; very free perspiration: feeling very comfortable when not annoyed by the cough.

Reduced the quinine to grs. i every four hours; gave an expectorant; applied croton oil over the throat and lungs; continued beef essence and brandy.

Thursday, 18th, ten A. M. Patient has had no fever since Wednesday night; some relish for food; cough still troublesome, but expectorating freely; continued the expectorant and quinine, nourishing diet, animal broth, and dismissed the case.

S. B. Junkin.

